# Study on the MRI features of normal postoperative glenoid labrum compared to recurrent tears

**DOI:** 10.6026/9732063002001823

**Published:** 2024-12-31

**Authors:** Saurabh Gangwar

**Affiliations:** 1 Department of Radiology, Rajshree Medical Research Institute & Hospital Bareilly, Uttar Pradesh, India

**Keywords:** Glenoid labrum, MR arthrogram, magnetic resonance imaging (MRI), recurrent glenoid labral tears

## Abstract

The study evaluates the effectiveness of MRI and MR arthrograms in detecting recurrent glenoid labral tears, highlighting MRI's ability to visualize soft tissues and assess postoperative repair integrity, crucial for diagnosing labral injuries and ensuring appropriate treatment. The study included 25 patients (72% male, 28% female) with recurrent shoulder repair. Recurrent labral tears were observed in 14 patients on MR arthrogram with 81-91% sensitivity and 76-86% specificity based on age. In 12% of patients, paralabral cysts were observed. Overhead activity was present in 44% of patients and most frequently in males under 30. Recurrent labral tear is seen in most of the patients with MRI imaging. The study found that MRI and MR arthrogram are useful diagnostic instruments with comparatively high sensitivity and specificity for detecting recurrent labral tears in postoperative patients, especially in patients between 35-40 years. This retrospective study evaluates the diagnostic accuracy of MR arthrograms in detecting recurrent glenoid labral tears after surgery, analyzing sensitivity, specificity, demographics, recurrence causes and secondary findings.

## Background:

The fibro cartilaginous glenoid labrum deepens the glenoid fossa and raises the articular surface of the glenohumeral joint, both of which improve joint stability. It helps to attach glen humeral ligaments and long head of the biceps brachii tendon (LHBT). Common shoulder problems, which are thought to be predominantly secondary to glenoid labral tears, include catching, instability and popping. Often, these can be relieved by surgical repair of the tears. These postoperative patients may experience recurrence injuries or chronic symptoms in up to 20% of cases. Additionally, MRI imaging can be considered in such patients after labral surgery [1, 2-3]. On MR arthrogram, the most reliable features for diagnosing a recurrent labral tear were signal intensity matching the adjacent glenoid labrum and a significantly reduced labral size. Signal confined to the anterosuperior quadrant beneath the labrum may represent a normal finding. Additionally, the presence of a paralabral cyst proved to be a highly sensitive secondary indicator of a recurrent tear [4]. Open or arthroscopic methods can carry out surgeries. For rotator cuff repairs and in acromioplasty, surgical intervention demands elevation of the deltoid from the acromion. Arthroscopy, on the other hand, is performed through minor incisions with arthroscopic instruments implanted. Some advantages of the open surgical approaches include direct visualization in acromioplasty and cuff repair; it can be done easily without requiring specialized equipment with long-term results. Two of its major disadvantages are the incapacity to reach intra-articular abnormalities other than extremely extensive rotator cuff tears and the need to separate the deltoid muscle, raising perioperative morbidity. The use of arthroscopic procedures is growing due to its advantages over open surgery, such as smaller scars, less severe pain, fewer complications and faster postoperative rehabilitation [5, 6]. This requires a clear understanding of common abnormalities in MR imaging of the postoperative shoulder since these surgical procedures are increasingly used to manage internal derangements of the glenohumeral joint. The decision to use MR arthrography or MR imaging in assessing glenoid labrum lesions appears to be based on presentation features. Intrinsic MRI imaging contrast infringement by effusion or changes in soft tissues is more characteristically seen in the acute stage or with more severe and unstable lesions of the pathologic conditions; hence, the diagnosis and characterization are non-invasive [7]. On the other hand, MR arthrography is more frequently necessary for patients with persistent symptoms or a pathologic abnormality that is thought to be milder based on clinical assessment [8]. Under ideal conditions, the labrum is best evaluated on standard MR imaging without arthrography with fluid-sensitive sequences in three planes. The same images are used to obtain fat-saturated T2-weighted sequences to assess the rotator cuff tendons more accurately. The oblique coronal and axial planes are the most useful for assessing the labrum [9]. In normal MRI imaging, anatomic variations have been found to occur frequently in the anterosuperior region and superior labrum, with an incidence of 13.5%, as found by researchers with significant variation [10,11]. Most common labral variants occur between the 11 and 3 o'clock locations (Figure 2a); the labrum should be firmly attached to the glenoid below the 3 o'clock location. However, various researchers have reported conflicting conclusions indicating labral variations can extend posterior to the LHBT origin at the supraglenoid tubercle and below the 3 o'clock position [11, 12-13]. Many studies have shown that MR imaging can identify labral tears with sensitivity ranging from 44% [14] to 95% [15]. Therefore, it is of interest to evaluate the appearance of the glenoid labrum on MRI after surgery, focusing on distinguishing recurrences from what has been termed normal postoperative changes.

## Methods:

## Study design and population:

**Figure 1:**
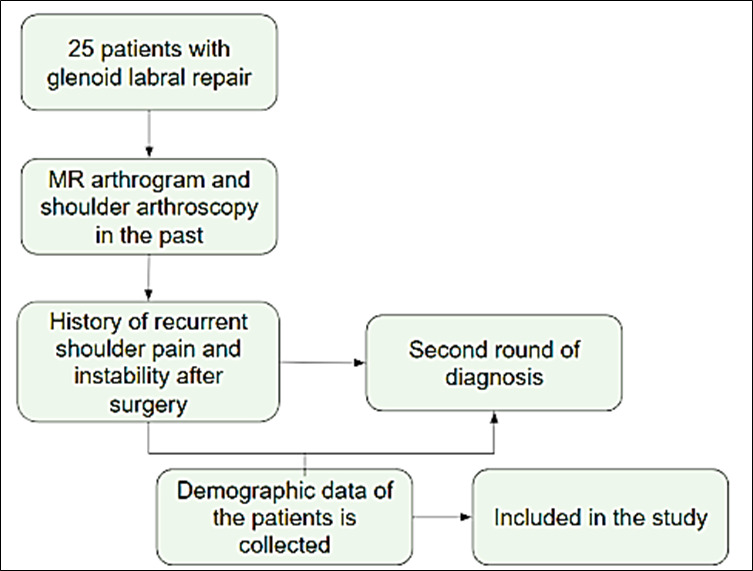
Flowchart of study design

This retrospective study assessed the accuracy of the MR arthrogram in diagnosing recurrent glenoid labral tears. A total of 25 patients who underwent glenoid labral repair followed by MR arthrogram in the past and repeat shoulder arthroscopy were included in the study (Figure 1). All the included patients gave a history of recurrent shoulder pain or instability after the surgery and received a second round of diagnostic evaluation and surgery. Patients' demographic and clinical data were collected including age, gender and involvement of the dominant arm.

## Inclusion and exclusion criteria:

Patients who underwent glenoid labral repair followed by MR arthrogram in the past and repeated shoulder arthroscopy after MR arthrogram with complete documented previous history were included in the study. Patients who did not follow up after the MR arthrogram and had incomplete medical data were excluded from the study.

## Status of labrum:

[1] Normal

[2] Irregular

[3] Torn

## Statistical analysis:

The accuracy of the MR arthrogram in diagnosing recurrent labral tears was evaluated by comparing the surgical and imaging findings. Overall rates of agreement, specificity and sensitivity were calculated using the results of the MR arthrogram. The percentage of recurrent labral tears that the MR arthrogram correctly diagnosed was known as sensitivity, while the percentage of correctly identified cases that did not have a recurrent tear was known as specificity. Discrepant cases were noted where the results of MR arthrogram varied from surgical results.

## Evaluation of MR arthrogram:

Two blinded radiologists review the MR arthrogram of every patient to assess the presence of recurrent labral tears. For every patient, the following results were noted.

## Paralabral cysts:

Present or absent

## Sture anchors:

It has been demonstrated that the glenoid suture anchors may account for any imaging alterations after surgery. Based on the glenoid labrum appearance on the MR arthrogram, the findings were divided into 3 groups: irregular, torn and normal.

## Evaluation of operative report:

The reports of repeat arthroscopy were reviewed for the findings of the glenoid labrum. It is categorized as irregular, torn, or normal, similar to MR arthrogram. These findings are used as a standard for comparing with the results of MR arthrogram.

## Secondary findings:

The paralabral cysts were assessed as secondary markers for the recurrent labral tears and the relation between labral tears and paralabral cysts was evaluated. The sensitivity in the prediction of a tear was calculated.

## Collection of data:

Age, gender and history of previous shoulder injuries were recorded to evaluate the correlation between these variables and the risk of recurrent tears. To do additional research on the possible reasons and mechanisms of labral tear recurrence, this data was utilized to stratify the patients by gender and age groups (Figure 2b) shows a typical tear at the glenoid labrum.

## Ethical considerations:

The study was conducted based on ethical guidelines and the confidentiality of the patient was maintained throughout the review. As this was a retrospective study, no additional intervention or risks were involved.

## Results:

Table 1 lists the demographic details of the 25 patients in the research. According to the age distribution, 32% of participants were between the ages of 30 and 35, while the majority (40%) were between the ages of 35 and 40. The gender distribution showed that 72% of the participants were men. Regarding dominant arm involvement, 60% of patients said their dominant arm was affected. Every subject had prior labral repair and only 12% of patients had paralabral cysts, whereas 88% did not. Table 2 reveals the patients' primary causes of recurrent glenoid labral tears. Overhead activity was the most common cause, accounting for 44% (11 patients). Trauma contributed to 32% of the cases (8 patients), while 24% (6 patients) experienced recurrent tears due to overuse or chronic strain. Table 3 examines the association between tear mechanism, age range and gender. Overhead activity was most prevalent in younger patients under 30, affecting 8 males and 3 females. Trauma was the primary tear mechanism in patients aged 30 to 40 years, impacting 5 males and 1 female. In the over-40 age group, chronic strain was the most common cause, with 2 males and 1 female affected. Table 4 details the MR arthrogram findings based on gender. Among the 18 male patients, 11 were found to have recurrent labral tears, 5 exhibited irregular labrum and 2 had normal labrum findings. Among the 7 female patients, 3 were diagnosed with recurrent labral tears, 3 had irregular labrum and 1 presented with a normal labrum. Table 5 focuses on the sensitivity and specificity of MR arthrogram findings based on age. In patients under 30 years, the sensitivity of the MR arthrogram was high at 91%, with a specificity of 81%. For patients aged 30 to 40, the sensitivity was slightly lower at 86%, with a specificity of 76%. In patients over 40 years, sensitivity dropped to 81%, but specificity increased to 86%. Table 6 provides insights into the presence of paralabral cysts based on age and gender. Paralabral cysts were absent in patients under 30 years old. The majority, or 66.6%, of cysts were found in the 30 to 40-year age range, predominantly in males (66.6%), while 33.3% were found in females in the over-40 age group. Table 7 compares operative reports with MRI diagnoses to assess MRI accuracy. In cases of recurrent labral tears, MRI diagnoses aligned with operative findings in 17 cases but were discrepant in 4. MRI findings matched operative reports for irregular labrum in 8 cases but differed in 3. There was also one instance where MRI indicated a normal labrum, but surgery revealed otherwise, highlighting occasional discrepancies in MRI diagnosis for labral abnormalities.

## Discussion:

The normal labrum looks hypo intense in MR imaging because of its short T2 relaxation time, primarily thought to result from the homogeneous nature of fibrocartilage. However, the labrum was proven neither entirely fibro cartilaginous nor homogeneous, increasing signal intensity in linear or globular regions, especially on images weighted intermediate and older people [16, 17]. The high signal intensity of the labrum is of uncertain clinical significance and may be considered a normal variant or an early degenerative or posttraumatic change [8]. Intermediate signal intensity may also be present at the chondrolabral junction, which represents the transitional zone of fibrocartilage and should not be confused with a labral tear [18]. Postoperative redundancy or thickening of the joint capsule may occur due to capsular plication with labral repair. This is best viewed as a low-signal-intensity structure adjacent to the labrum and is considered a normal postoperative finding [19]. In MR findings, after labral debridement, a non-existent labrum or a diminished labrum with reactive productive alterations of the glenoid rim nearby are often seen [20]. The routine postoperative MRI appearance following a Bank art reconstruction is a thickening of the anterior joint capsule and labrum, with labral fragmentation that is reattached to the rim [21]. MR imaging after a Latarjet procedure should demonstrate a well-attached bone block in the anteroinferior glenoid. The subscapular is muscle commonly has scar tissue present, representing the expected outcome of the formation of the intramuscular split [22]. Remplissage is classically a transfer of the posterior joint capsule and infraspinatus tendon into a significant Hill-Sachs defect and typically occurs with suture anchors [23]. Inferior capsular movement on postoperative MRI will produce an artefact in the magnetic susceptibility of the capsule. The capsule needs to be thick, continuous and watertight. The anterior capsule is redundant and scarred and will present as a focal mass effect on the subscapular is tendon articular surface [19]. Superior labrum anteroposterior (SLAP) tear is a tear of the labrum that extends anterior or posterior to the bicep's origin. The SLAP was grouped into four kinds [24]. Type IV lesions often require a biceps tenodesis due to the tear's involvement in the biceps tendon insertion [25]. The labral contour of SLAP repairs should be smooth and no fluid or contrast should be seen as accumulating within the labral material. Often, the material of the labrum secondary contains signals with variable intensity. Results should not be confused with a recurrent SLAP tear due to granulation or scar tissue and surgical debris impregnated within the labral substance at the site of the previous tear [26]. The absence of co-existent pathology in the adjacent glenoid rim and capsule ligamentous structures helps distinguish between surgical signal changes and persistent tears. Post-SLAP repair, recurrent labral tears are an imaging finding on MRI as a roughened labral surface or a contrast or fluid signal appearing at the base or substance of the repaired superior labrum [26]. An abnormally loose suture anchor may also suggest re-tear and can be free-floating and defined by contrast solution [1]. Postoperative changes of biceps tenotomy on MRI often include the failure to visualize the intra-articular part of the long head of the biceps tendon, which active changes at the supraglenoid tubercle may accompany. Screws, anchors, or sutures may give an illusion of foci of susceptibility artefact over the site of attachment of the distal part of the biceps tendon to the humeral head or proximal humeral shaft [19, 27]. A recurrent tear in the glenoid labrum is characterized by a disruption or discontinuity of the labral tissue, often seen as a focal defect on T2-weighted images. It is often accompanied by fluid-sensitive signal changes, such as high intensity on fluid-sensitive sequences [28]. In postoperative MR imaging, gadolinium contrast enhancement can highlight recurrent tears by showing leakage into abnormal spaces [28]. A labral tear that fails to heal properly may appear detached from the glenoid rim. Chronic recurrent tears may lead to bony glenoid or humeral head changes, suggesting chronic instability [29]. Close collaboration between radiologists and clinicians and an understanding of surgical history and patient symptoms is crucial to diagnose postoperative shoulder issues accurately.

## Conclusion:

The study's main findings highlight a high prevalence of recurrent glenoid labral tears among males (72%) and patients aged 35 to 40 (40%). Overhead activity is the leading cause of recurrence, particularly among younger patients, while trauma is the most frequent cause for those aged 30-40. The MR arthrogram proves highly sensitive (91%) and specific (81%) in younger patients but shows variable accuracy with age. Operative findings largely confirm MRI diagnoses, though discrepancies exist, especially in identifying recurrent labral tears. Paralabral cysts are rare; appearing primarily in males aged 30-40. This study highlights MRI and MR arthrogram as effective tools for detecting recurrent labral tears; especially in patients aged 35-40, after shoulder surgery. Despite MRI's diagnostic value, occasional discrepancies with surgical findings suggest cautious interpretation and, if needed, surgical follow-up. MR arthrogram remains significant in distinguishing recurrence from normal healing.

## Funding:

No funding was received for this research.

## Figures and Tables

**Figure 2a:**
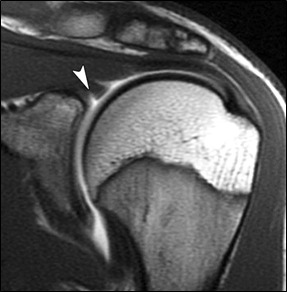
High signal intensity extending medially and follow the contour of glenoid cartilage and having smooth margin-s/o Sub labral recess-Normal variant. High signal intensity seen in superior labrum with fraying of margin-s/o Slap tear

**Figure 2b:**
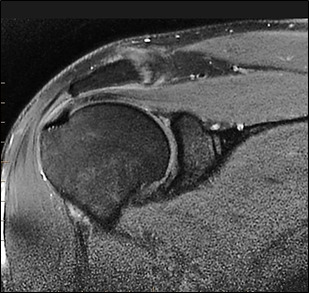
High signal intensity seen in superior labrum with fraying of margin-s/o Slap tear

**Table 1:** Demographic characteristics of patients in this study

**Characteristics**	**Number of patients (n= 25)**	**Percentage**
**Age (years)**		
30-35	8	32%
35-40	17	40%
**Gender**		
Female	7	28%
Male	18	72%
**Dominant arm involved**		
Yes	15	60%
No	10	40%
**Previous labral repair**		
Yes	25	100%
No	0	0
**Paralabral cyst**		
Yes	3	12%
No	22	88%

**Table 2:** Cause of recurrent glenoid labral tear

**Cause**	**Number of patients (n= 25)**	**Percentage**
Overhead activity	11	44%
Trauma	8	32%
Overuse or chronic strain	6	24%

**Table 3:** Tear mechanism associated with age and gender

**Mechanism**	**Age range**	**Male (n=18)**	**Female (n=7)**
Overhead activity	< 30	8	3
Trauma	30 - 40	5	1
Overuse or chronic strain	> 40	2	1

**Table 4:** MR arthrogram distribution based on gender

**MR arthrogram findings**	**Male (n=18)**	**Female (n=7)**
Recurrent labral tear	11	3
Irregular labrum	5	3
Normal labrum	2	1

**Table 5:** Specificity and sensitivity of MR arthrogram based on age

**Age range**	**Sensitivity (%)**	**Specificity (%)**
< 30	91%	81%
30 - 40	86%	76%
> 40	81%	86%

**Table 6:** Paralabral cyst presence based on gender and age

**Characteristic**	**Presence of paralabral cyst (n=3)**	**Percentage (%)**
Age (years)		
< 30	0	0
30 - 40	2	66.60%
> 40	1	33.30%
Gender		
Male	2	66.60%
Female	1	33.30%

**Table 7:** Relationship between Operative Report and MRI Diagnosis

**MR diagnosis**	**Agreed with operative report (n=25)**	**Discrepant with operative report (n=8)**
Recurrent labral tear	17	4
Irregular labrum	8	3
Normal labrum	0	1
